# Ins and Outs of Heat Shock Proteins in Colorectal Carcinoma: Its Role in Carcinogenesis and Therapeutic Perspectives

**DOI:** 10.3390/cells10112862

**Published:** 2021-10-24

**Authors:** Batoul Abi Zamer, Waseem El-Huneidi, Mohamed Ahmed Eladl, Jibran Sualeh Muhammad

**Affiliations:** 1 Department of Basic Medical Sciences, College of Medicine, University of Sharjah, Sharjah 27272, United Arab Emirates; batoul.abizamer@gmail.com (B.A.Z.); welhuneidi@sharjah.ac.ae (W.E.-H.); meladl@sharjah.ac.ae (M.A.E.); 2 Sharjah Institute for Medical Research, University of Sharjah, Sharjah 27272, United Arab Emirates

**Keywords:** heat shock proteins, colorectal carcinoma, chaperones, theranostics

## Abstract

Cancer cells can reprogram their metabolic activities and undergo uncontrolled proliferation by utilizing the power of heat shock proteins (HSPs). HSPs are highly conserved chaperones that facilitate the folding of intracellular proteins under stress. Constitutively, HSPs are expressed at low levels, but their expression upregulates in response to a wide variety of insults, including anticancer drugs, allowing cancer cells to develop chemoresistance. In recent years, several researchers have reported that HSPs could be an important therapeutic target in difficult-to-treat cancers such as colorectal carcinoma (CRC). Worldwide, CRC is the second most common type of cancer and the second leading cause of cancer-related deaths. The molecular complexity of CRC and the coexisting inflammatory conditions present a significant obstacle to developing effective treatment. Recently, considerable progress has been made in enhancing our understanding of the role of HSPs in CRC pathogenesis. Moreover, novel therapeutic strategies targeting HSPs, either alone or in combination with other anticancer agents, have been reported. Herein, we present an overview of the functional mechanisms and the diagnostic and prognostic potential of HSPs in CRC. We also discuss emerging anti-CRC strategies based on targeting HSPs.

## 1. Introduction

As a defense mechanism, normal cells under stressful conditions such as hyperthermia, starvation, or oxidative stress start synthesizing a group of molecular chaperones known as heat shock proteins (HSPs). HSPs serve as chaperones in protein maturation, refolding, transportation, and degradation to regulate cellular proliferation and differentiation [1]. They are categorized according to six main groups based on their molecular weight: small HSPs (sHSPs), large HSPs, HSP40s, HSP60s, HSP70s, and HSP90s [2]. HSPs were first discovered in 1962 when heat shock caused chromosomal puffs in Drosophila melanogaster larvae [3]. Following that breakthrough, researchers have reported that HSPs are ubiquitously expressed by all organisms and are highly conserved at both gene and protein levels [2,4], suggesting the importance of their function in normal cells. 

The overexpression of HSPs under stressful stimuli is a characteristic of normal cells. Cancer cells, however, are addicted to chaperones and constitutively upregulate their expression to inhibit cell apoptosis and promote abnormal cell growth [5]. The overexpression of HSPs has been extensively linked to colorectal cancer (CRC) progression [6,7]. HSPs are now considered oncogenic regulators, and studies have extensively reported their use as biomarkers in CRC diagnosis and progression [7]. Additionally, elevated HSP expression, of HSP47 and HSP110 serves as a good predictive marker for lymph node metastasis of CRC [8,9]. Understanding the functional mechanisms by which HSPs aid cancer progression and development can open the door for novel biomarkers and targeted therapies for CRC. 

Various anticancer therapies targeting CRC have shown to select the expression of HSPs as a tolerance mechanism of cancer cells to maintain their growth and survival [10,11,12] (graphical abstract); therefore, cancer cells develop resistance to chemotherapeutic treatments by overexpressing HSPs. For this reason, some anticancer treatments are now designed to inhibit HSP activity and have shown promising preclinical and clinical outcomes in CRC treatment [13,14]. The pivotal role of HSPs in CRC progression and metastasis makes them an attractive target for different anticancer therapies, mainly when used in combination therapy to improve their effectiveness and promote drug sensitization [15]. In this review, we discuss the functional role of HSPs in CRC progression and highlight the significance of HSPs as diagnostic and prognostic biomarkers and emerging anti-CRC therapies that target HSPs in CRC.

## 2. Functional Mechanisms of HSPs in CRC

In CRC cells, HSPs are overexpressed to protect the cellular proteins from degradation under stressful conditions [16]; hence, overexpression is almost always associated with an unfavorable prognosis and poor overall survival (OS). Various HSPs have been reported to promote CRC progression by mediating different cell growth-related pathways and molecules in CRC cells [17,18] (Table 1). There are two main types of HSPs based on how they regulate the folding of proteins. HSP90, HSP70, and sHSPs are categorized as one type requiring direct interaction with the targeted proteins to perform their function, whereas the second type, including HSP60, forms complexes with other proteins, like folding chambers that provide proper folding for unfolded proteins [16].

### 2.1. HSP90 Family

#### 2.1.1. Role in CRC

HSP90s are a highly conserved selective molecular chaperone family that is overexpressed in different cancers, and they are considered oncogenes in CRC [41]. These chaperones selectively stabilize and activate a group of client proteins [42]. Many HSP90 client proteins have a role in cancer biology because they regulate essential cellular functions such as the cell cycle, apoptosis, and proliferation. Examples of these proteins are p53 missense mutants (mutp53), Akt, ErbB2, hypoxia-inducible factor-1 alpha (HIF-1α), and BCR-ABL [43]. One of the mechanisms by which HSP90 promotes CRC is stabilizing mutp53 via inhibition of Mdm2 and CHIP E3 ubiquitin ligases that usually degrade mutp53 [19]. The mutp53 mutation causes loss of P53 and constitutes a gain of function mutation that promotes cancer progression when stabilized by HSP90 [44]. Notably, the inhibition of HSP90 caused CRC cell depletion due to mutp53 and cancer regression in a mouse model [19]. HIF-1α is an oncogenic transcription factor that, following oxygen depletion, is upregulated to increase the transcription of the primary genes involved in cell proliferation. HIF-1α also causes the metabolic switching of CRC from oxidative phosphorylation (OXPHOS) to anaerobic glycolysis—a phenomenon known as the “*Warburg effect*” [45]. Recently, a study reported that the upregulation of HSP90 in CRC mediated F-FDG accumulation, which is a common phenomenon of the *“Warburg effect”* [20]. Additionally, the same study suggested that this event might have been caused by the upregulation of HIF-1 α and F-FDG binding to HSP90. Other recently identified HSP90 client proteins include the high mobility group AT-hook 2 (HMGA2) and E3 ubiquitin-protein ligase Hakai [21,41]. HMGA2 is a non-histone transcription factor that binds to chromatin and regulates the expression of other proteins. When HSP90 was inhibited, HMGA2 was downregulated and inhibited CRC cell growth [21]. Of all the targets of Hakai proteins, the most studied one is E-cadherin. Inhibition of HSP90 causes accumulation of its target, E-cadherin, and subsequent inhibition of CRC metastasis [41]. 

HSP90 promotes its function with the help of partner proteins, such as cell division cycle 37 (Cdc37), stress-inducible protein 1 (Sti1), p23, and protein phosphatase-5 (PP5) [46]. Cdc37 is a co-chaperone that binds to HSP90 to form a complex with protein kinases, increasing their stability and enhancing their kinase activity [46]. When activated, Cdc37 activates one of the most oncogenic CRC signaling pathways, the mitogen-activated protein kinase (MAPK) pathway [46]. Besides cytoplasm and cell membrane localization, HSP90 is also secreted by cells into the extracellular matrices [43]. When secreted, HSP90 interacts with the cell surface protein CD91, which binds to other cell-surface proteins such as integrin. This interaction causes the subsequent activation of various signaling pathways to maintain cellular growth and inhibit apoptosis. Remarkably, short hairpin RNA (shRNA) targeting the integrins has been shown to inhibit the oncogenic activity of HSP90 and lead to subsequent CRC regression [43]. Additionally, secreted HSP90 promotes EMT and invasion of CRC by binding to TCF12. TCF12 is a member of the helix-loop-helix (HLH) family that, when overexpressed, downregulates E-cadherin to promote CRC invasion and metastasis [18].

Glucose-related protein 94 (GRP94) is also a member of the HSP90 family uniquely expressed on the ER, but it has a similar function to other HSPs in protein refolding and assembly under stress; therefore, GRP94 overexpression has been observed to play a protumor role in many cancers, including CRC. However, depending on the situation, GRP94 has also been reported to play an antitumor role in causing cancer inhibition by facilitating the activation and maturation of immune cells and overall immune response [23].

Tumor necrosis factor receptor-associated protein 1 (TRAP1) is another HSP90 chaperone mainly expressed in the mitochondrial matrix. TRAP1 has a different function from other HSPs in that it protects the mitochondria from the accumulation of ROS, which is a cancer event. Consequently, its upregulation has been detected in different cancers, including CRC [24]. The mechanism by which TRAP1 promotes CRC is not fully understood, but its biological significance seems vital, and its role in CRC development requires further analysis.

#### 2.1.2. Diagnostic and Prognostic Values

HSP90 plays a significant role in cellular proliferation and survival, and its overexpression has been reported to be strongly associated with CRC [41]; therefore, the expression of HSP90 was assessed in CRC patients to analyze its prognostic significance. Immunohistochemistry on 99 CRC tissue samples and 81 normal tissues showed that elevated expression of HSP90 was associated with poor prognosis in CRC patients. HS90 was also identified as an independent risk factor for OS [22]. However, HSP90 has two different isoforms—HSP90α and HSP90β—and HSP90β had no prognostic impact on CRC patients [47]. Furthermore, TRAP1 was identified as a prognostic marker for CRC, and its overexpression was associated with CRC invasion and a significant reduction in disease-specific survival [24]. Finally, the overexpression of GRP94 was also found to be significantly associated with an increased overall tumor size and pT stage in CRC patients [23]. GRP94 is known to be associated with good CRC prognoses [23]. The use of GRP94 as an effective or ineffective prognostic marker is mainly affected by its function as an oncogene or tumor-suppressor gene based on the tumor microenvironment. Studies involving the use of HSPs as prognostic markers would provide a greater understanding of the natural history of CRC, allowing the development of more therapy options. 

### 2.2. HSP70

#### 2.2.1. Role in CRC

HSP70 is a major component of the HSP family network that is usually expressed under stressful conditions [2,48]. HSP70 is ubiquitously expressed in humans and has various protein folding functions, including folding new proteins, refolding aggregated proteins, and preventing protein aggregation [49]. HSP70 is an ATP-mediated chaperone with two domains. Whereas its C-terminal domain promotes binding to proteins, its N-terminal domain is an ATPase that allows proper protein folding [16]. HSP70 is a family of 13 chaperones, almost half of which have been reported to play a role in various cancers [2]. The HSP70 family includes HSP70, HSP70-2, HSC70, glucose-regulated protein 75 (GRP75), and GRP78, expressed in different cell compartments, such as the cell surface, cytosol, and mitochondria [2]. 

After analyzing 200 CRC samples, HSP70-2—a cancer-testis (CT) antigen—was reported to be significantly and positively associated with CRC independently of the tumor stage. Additionally, in the same study, silencing of the *HSP70-2* gene caused significant regression of CRC malignancy [50]. Furthermore, GRP75 (also known as mortalin) was observed to promote CRC cell proliferation through P53 retention [28]. In other words, mortalin can bind to P53 and cause its sequestration in the cytoplasm to inhibit its tumor suppressor properties. Additionally, GRP78 is a particular oncogene in the BRAFV600E CRC subtype [29]. Analyzing the genetic profile associated with this subtype revealed that different biological processes were altered, including the activation of the UPR. HSP GRP78 mediates proper BRAFMT CRC cell proliferation and stability by regulating UPR [29]. Additionally, mortalin activates the Wnt/β-catenin pathway to promote cell proliferation and EMT transition in CRC cells [26]. A novel function of HSP70 has recently proved to be involved in CRC progression by activating oncogenic pathways such as the RTK signaling pathway. These signals stabilize β-catenin and promote its accumulation, which, in turn, activates its targets to mediate CRC development [25]. 

At the epigenetic level, HSP70 was shown to be a primary target for lncRNA RP11-462C24.1 (RP11-462C24.1) in CRC cell lines. Overexpression of RP11-462C24.1 inhibited cancer growth and metastasis and reduced the HSP70 level in CRC [51]. However, this was the only study to discuss the effect of epigenetic alteration on CRC, and further studies are warranted to understand the HSP70 mechanism in CRC.

#### 2.2.2. Diagnostic and Prognostic Values

HSP70 maintains the activity of several client proteins involved in cancer growth and metastasis [52]; thus, elevated expression of HSP70 is widely reported in different cancers, including CRC. This overexpression correlates with poor OS, invasion, and metastasis, and resistance to chemotherapy in CRC. The HSP70 member, mortalin, is associated with poor OS and may indicate an adverse prognosis for CRC patients [26]. Interestingly, according to recent studies, the Hsp70 family is the worst independent prognostic biomarker for primary CRC [27].

HSP70 was also shown to be significantly overexpressed in CRC patients compared to a control group, and this overexpression was associated with high mortality. This study proved the benefit of HSP70 as a prognostic marker for CRC with no metastasis, independently of the tumor stage [53]. However, a study using 232 CRC patients found that HSP70 serum levels were significantly associated with the tumor stage and poor OS in those patients, further proving the significance of HSP70 as a prognostic marker for CRC [54]. Additionally, the study used TCGA database analysis to investigate the importance of *Hsp70* genes as prognostic markers in 438 CRC patients. The reduced expression of 4 out of 13 tested *HSP70* genes, including *HSPA1A*, *HSPA1B*, and *HSPA1L,* showed a significant association with improved OS in CRC patients. This study proved these *Hsp70* genes as latent prognostic markers in CRC patients [27]. Moreover, the overexpression of stress-induced phosphoprotein 1 (STIP1)—a protein that links HSP70 to HSP90—was observed in CRC tissue compared to normal tissue. This overexpression was associated with the tumor stage and worse OS and, thus, proved to be helpful as an independent prognostic marker in patients with CRC.

### 2.3. sHSPs Family

#### 2.3.1. Role in CRC

sHSPs are a highly conserved family of ten chaperones with molecular sizes ranging from 15–30 kDa. Their unique feature is the α- crystalline domain, a string of homolog 80 amino-acid residues [34]. HSP27 (also called HSPB1) is the most well-studied chaperone of the sHSPs family; it is expressed mainly in the cytosol, and its functional mechanism in CRC is well known [31,55]. HSP27 is an ATP-independent chaperone that does not hydrolyze ATP for polypeptide refolding [16]. Instead, HSP27 forms large oligomers regulated by phosphorylation in three serine residues—Ser15, Ser78, Ser82—and a threonine residue (Thr143) [2]. HSP27 plays a significant role in inhibiting apoptosis and mediating cellular proliferation and metastasis; thus, its ectopic expression has been significantly observed in CRC [31]. Moreover, it promotes CRC EMT and metastasis [30]. The knockdown of HSP27 inhibited CRC cell proliferation, migration, and differentiation [31], which was thought to be caused by the inhibition of the G2\M transition phase due to cell cycle-associated molecule downregulation. In addition, Ca^2+^ influx—an oncogenic event—is regulated by HSP27 in CRC. This correlation is based on a selective effect of HSP27 on Stromal Interaction Molecule 1 (STIM1)—a calcium sensor. It has been reported that HSP27 promotes CRC progression via the STIM1-mediated Ca^2+^ influx pathway [31]. HSP27 has been reported to play a significant role in regulating the *“Warburg effect”* in CRC [32]. The accumulation of methylglyoxal (MGO) is one of the hallmarks of CRC cells following upregulated aerobic glycolysis. MGO has a glycation ability that overuses glucose by glycating various molecules, including lipids, DNA, and proteins, including HSP27. Glycation of HSP27 enhances its stability and activity in protein folding; thus, modified MGO-Hsp27 has a remarkable ability to promote CRC cell proliferation and metastasis [32]. HSP27 is regulated at the epigenetic level. The antitumor microRNA-577 has been reported to inhibit CRC progression by sponging different oncogenic long noncoding RNA (lncRNA), such as lncRNA LINC00520 and lncRNA differentiation antagonizing non-protein coding RNA (DANCR) [56,57,58]. Both LINC00520 and DANCR have been shown to promote the ectopic expression of HSP27 in CRC [57,58]. Additionally, the lncRNA taurine-upregulated gene 1 (TUG1) upregulates HSP27 and enhances its oncogenic activity [59]. However, the ectopic expression of miR-214 has been reported to downregulate HSP27 and its activity in CRC development [60]. 

Another sHSP known as heat shock protein beta 3 (HSPB3) has recently been shown to play a role in CRC progression and metastasis [34]. HSPB3 is expressed in all types of organisms but with higher expression in skeletal and smooth muscles. HSPB3 forms oligomers with the other sHSPs known as HSPB2 to promote muscle cell differentiation [34]. The mechanism by which HSPB3 is involved in CRC progression has yet to be elucidated; therefore, it is crucial to understand the carcinogenic role of HSPB3 in CRC to allow for a better understanding of the disease mechanism. 

Alpha B-crystallin (CRYAB)—also known as HSPB5—is one of the sHSPs expressed in several body organs such as the heart, brain, and skeletal muscles [35]. CRYAB protective and therapeutic roles have been extensively reported in different inflammatory diseases [35]. Using two different CRC cell lines—HT29 and Caco-2—a study found that CRYAB manipulated the responses of these cells towards inflammatory stimuli, inhibiting the NF-κB. This inhibition caused the downregulation of essential pro-inflammatory cytokines [35]. However, CRYAB’s exact role in CRC is still under-studied, which could help identify different therapeutic targets.

#### 2.3.2. Diagnostic and Prognostic Values

Different single nucleotide polymorphisms (SNPs) are now used as prognostic and diagnostic markers for CRC since some are associated with inhibited metastasis and others with enhanced metastasis. SNPs are now commonly used as biomarkers to assess the role of HSPs in CRC. These SNPs could upregulate or downregulate the HSP function in protein folding and therefore play a critical role as disease biomarkers; for example, the HSPB1 rs2070804 SNP has been associated with the depth of the primary tumor depth in CRC, but not appropriate for indicating CRC metastasis or recurrence [33]. Furthermore, the expression of sHSPs in cancer-associated fibroblasts (CAFs) has been reported as an important prognostic factor since the stromal expression of Hsp27 reduced recurrence-free survival in patients with CRC following pulmonary metastasis [61].

The prognostic value of CRYAB has been reported in CRC [36]. A study of 111 CRC patients found that CRYAB was strongly linked to the tumor grade, with poorly differentiated G3 tumors having the lowest CRYAB expression. This study demonstrated that CRYAB is a potential prognostic biomarker for assessing CRC [36]. Using multivariable Cox regression to assess 188 colorectal adenocarcinoma samples compared to 68 noncancerous ones, another study demonstrated the prognostic value of HSPB3 in CRC [34]. The upregulation of HSPB3 expression in CRC patients was associated with poor RFS and OS regardless of their tumor stage or grade; therefore, this study suggested that HSPB3 is an excellent prognostic marker for CRC, regardless of clinicopathological prognosticators [34]. Understanding the role of HSP27 as a prognostic marker for CRC would broaden personalized treatments, thereby enhancing overall CRC outcomes.

### 2.4. HSP60

#### 2.4.1. Role in CRC

HSP60 is a ubiquitous ATP-dependent mitochondrial chaperone expressed in all tissue types and implicated in different physiological processes. It is responsible for the refolding, stabilization, and translocation of essential mitochondrial proteins [38]. Unlike other HSPs, the data showing the link between HSP60 expression and cancer are controversial and inconclusive [62]; for instance, ongoing research is still considering whether HSP60 is an oncogene or tumor suppressor in hepatocellular carcinoma (HCC) [62]. Additionally, HSP60 expression is downregulated in some types of cancers and upregulated in others; for example, HSP60 is downregulated in cancers such as clear cell renal cell carcinoma (ccRCC), and its overexpression reduces cell proliferation, whereas its knockdown activates the *“Warburg effect*” in CRC [63,64].

The overexpression of HSP60 has been linked to different pathological conditions and cancers, including ovarian cancer, prostate cancer, and CRC [65,66,67]. In CRC, HSP60 is regulated by the tumor-suppressor gene *IGFBP7*. HSP60 is a downstream protein of IGFBP7, and the antitumor activity of *IGFBP7* is caused by the inhibition of HSP60 activity [37]. Furthermore, a study reported that CRC cells require elevated HSP60 expression for proper tumor development, and the knockdown of HSP60 reduced the proliferation of CRC cells following both in vitro and in vivo experiments. HSP60 knockdown disrupts most of the mitochondrial proteins in CRC cells, causing adenine accumulation, which, in turn, activates the AMPK and inhibits cell proliferation [38]. Thus, suggesting an oncogenic role of HSP60 and affecting the mitochondrial homeostasis in CRC progression, allowing the use of HSP60 as a therapeutic target for CRC.

#### 2.4.2. Diagnostic and Prognostic Values

Following the identification of HSP60 as an oncogene in CRC, the research community demonstrated the value of using HSP60 as a prognostic marker for CRC. In 2011, Hamelin et al. showed for the first time that HSP60 was overexpressed in 112 patients with CRC compared to 90 normal controls. They reported that HSP60 might serve as a novel prognostic marker for late-stage CRC, but further studies are warranted [39]. Later, a study of 97 patients and 79 healthy controls identified HSP60 as an effective marker for metastatic CRC. Additionally, HSP60 serum levels were reported as an excellent prognostic marker for liver metastasis in CRC patients [68]. Notably, serum levels are mostly preferred for diagnostic purposes and are less invasive than collecting solid biopsy samples. Additionally, HSP60 proved to be an early diagnostic marker for different digestive cancers, including CRC [69]. All these studies proved the significant value of using HSP60 in CRC, but further studies are warranted for its use in other types of cancers.

### 2.5. HSP110

#### 2.5.1. Role in CRC

HSP110 (also known as HSP105 or HSPH1) is the third most expressed chaperone in most of mammalian cells [70]. HSP110 is mainly localized in the cytosol and ER of eukaryotic cells, where it catalyzes nucleotide exchange factor for HSP70 to promote protein anti-aggregation and refolding [71]. Previous studies designated HSP110 as a member of the HSP70 family; however, advanced research has assigned HSP110 to a separate group due to its completely different and unique structure [72]. HSP110 plays a significant role in activating different cell proliferation pathways, explaining its association with many cancers. In CRC, HSP110 promotes cell proliferation by upregulation ofSTAT3 activity. Unlike other HSPs, HSP110 does not upregulate STAT3 expression; instead, it increases its phosphorylation and translocation to the nucleus [17]. Furthermore, HSP110 was found in a mutant form in CRC patients with microsatellite instability (MSI), known as HSP110DE9. The lack of exon 9 due to this mutation caused the aberrant production of HSP110 but inhibited its function [9]. Notably, patients with HSP110DE9 had a significant increase in immune cell infiltration. Furthermore, HSP110 was secreted by CRC cells, and this secretion was inhibited by HSP110DE9 overexpression affecting the inflammatory profile of macrophages. In other words, inhibiting extracellular HSP110 favors pro-inflammatory macrophages, whereas overexpression of extracellular HSP110 favors anti-inflammatory macrophages [40]. This study revealed the extracellular HSP110 as a new target for improving the immune response to cancer cells in CRC patients. 

#### 2.5.2. Diagnostic and Prognostic Values

Since the discovery of HSPs, their roles as prognostic makers have been extensively studied because of their significant role in maintaining cell growth under different stressful conditions; however, only a few studies with small patient samples have focused on HSP110 and its mutant form as prognostic markers for cancer patients. One of these studies proved that the overexpression of HSP110 is significantly associated with poor prognosis, reduced OS, and metastatic CRC [9]. HSP110DE9 inhibits the wild-type HSP110 function and is associated with better OS; therefore, HSP110DE9 has proved to be an effective prognostic marker for MSI in CRC patients [73]. Additionally, extracellular HSP110 was shown to affect the tumor microenvironment, favoring the anti-inflammatory macrophages, but HSP110DE9 inhibited HSP110 secretion and increased pro-inflammatory macrophages. This finding proved that HSP110DE9 was associated with a favorable CRC prognosis in CRC [40]. Further studies are warranted to examine the association of HSP110DE9 levels with the responses to different cancer therapies. 

## 3. Targeting HSPs for CRC Therapy

Increasing evidence has suggested HSPs as novel therapeutic targets for cancer therapy [74]. Because of their abnormal expression in cancer cells, the cells can produce proteins and thrive in the extremely harsh conditions of the cancer microenvironment [75]; thus, it is unsurprising that targeting HSPs with different inhibitors may overcome their oncogenic properties and exert a potent anticancer effect. Indeed, there has been increasing interest in identifying and developing inhibitors to target individual HSPs, attenuate their function, and inhibit cancer progression (Table 2).

### 3.1. HSP90 Inhibitors 

HSP90 inhibitors are the most extensively studied HSP inhibitors for cancer therapy [76]. HSP90 plays a significant role in cell proliferation, differentiation, and metastasis by interacting with and stabilizing major oncogenes to promote abnormal cancer cell growth [21]. Although the HSP90 family has four members with different client protein targets, they share the same structure. Because they are ATP-dependent chaperones on their N-terminal domains, they have ATP binding pockets. By targeting this domain, more than one HSP90 member can be inhibited using a single HSP90 inhibitor known as a pan-HSP90 inhibitor [77]. The first-in-class HSP90α inhibitor is 17-allylamino-17-demethoxygeldanamycin (17-AAG)—a geldanamycin derivative that specifically targets the ATP binding pocket of HSP90. 17-AAG inhibits the ATPase activity of HSP90 and consequently promotes degradation of HSP90 through proteasome mechanisms [78]. Following 17-AAG treatment, CRC cells were arrested at G_2_ due to a reduction in cyclin B1 levels.

Furthermore, 17-AAG downregulates STAT3 to induce apoptosis in CRC cells [78]. To date, more than 18 different HSP90 inhibitors have reached the clinical trial stage. Unfortunately, none have had sufficient efficacy to earn FDA approval [77] due to their elevated toxicity, since numerous HSP90 client proteins play an essential role in normal body development [79]. The research community is now looking for an alternative way to specifically target the oncogenic client proteins of HSP90, which may be achieved by targeting its protein-protein interaction (PPI) with other co-chaperones [79]. One example is targeting CdC37, which is a co-chaperone that explicitly allows for HSP90-kinase interactions. Instead of broadly targeting the ATPase activity of HSP90, targeting the HSP90-Cdc37 PPI would inhibit kinase maturation and allow for safe and specific anticancer treatment [79]. Different compounds are being tested for this interaction, showing promising results in both in vitro and in vivo experiments [79]. 

One of these compounds is the small-molecule inhibitor DDO-5936 [80]. DDO-5936 bound to Glu^47^ residue of HSP90 and disrupted the PPI with Cdc37 with subsequent inhibition of kinase client proteins of HSP90 in a CRC cell line. CRC cell proliferation was inhibited due to subsequent degradation of cyclin-dependent kinase 4 (Cdk4). Another limitation of HSP90 inhibitors is their export by ATP-binding cassette (ABC) transporters, making cancer cells resistant to these inhibitors [13]. An example of ABC transporters is P-glycoprotein (P-gp), which pumps foreign molecules outside cells to cause multidrug resistance (MDR); thus, it seems that dual-activity drugs targeting both HSP90 and P-gp have potent activity as anti-CRC therapies. For this purpose, eleven HSP90 inhibitors were tested to inhibit cancer growth and MDR caused by P-gp, and 3 out of the 11 tested compounds succeeded in inhibiting P-gp overexpression and MDR together with HSP90 in CRC cells. These compounds are potential anticancer treatments for overexpressing P-gp in CRC [13].

However, there are other mechanisms by which cancer cells can become resistant to HSP90 inhibitors, such as the target-independent activation of downstream proteins. This activation is mainly caused by mutations that change the target expression and activate an alternative pathway to produce the target [13]; therefore, HSP90 inhibitors potency may be improved when combined with other standard chemotherapies [81]. Following this suggestion, Wu et al. identified a series of drugs known as luoropyrimidin-2,4-dihydroxy-5-isopropylbenzamides using a combination of different chemotherapies with resorcinol and HSP90 inhibitors [82]. One of these compounds, 12c, was remarkably active in CRC cell lines, proving the role of this novel HSP90 inhibitor in CRC treatment. In another study, Moradi et al. combined 17-AAG with irradiation (Ir) and gold nanoparticle (GNP) therapies, revealing a potential anticancer treatment by inhibiting cell proliferation and the induction of apoptosis for CRC cells, which further proved the role of combination therapy [15]. It has also been reported that HSP90 inhibitors seem to have contradictory effects depending on the molecular subtype of CRC (CMS) [81], demonstrating a need for prior identification of patient CMS to improve the effectiveness of HSP90 inhibitors when combined with different administered chemotherapies. This combination may overcome resistance to HSP90 inhibitors and offer improved opportunities for CRC treatment. 

### 3.2. HSP70 Inhibitors

Unlike normal cells, HSP70 is overexpressed in cancer cells even under stressful conditions [6]; therefore, several anticancer therapies are now being designed to target HSP70 activity. Inhibitors of HSP70 are categorized according to three main groups: small-molecule inhibitors, peptide aptamers, and antibody-based therapy [2]. Several small-molecule inhibitors have been identified that target HSP70 activity in CRC. One of which is the natural compound epigallocatechin-3-gallate (EGCG), which has shown a promising antitumor effect on CRC in vitro and in vivo experiments. EGCG specifically targets the ATPase domain of Grp78 to promote its dimerization and, consequently, its inactivation [14]. Other small-molecule inhibitors that target HSP70 are quercetin and Kahweol [83,84]. CRC cells treated with either of these inhibitors exhibited reduced expression of HSP70 and a significant reduction in tumor growth. HSP70 can also be inhibited indirectly by targeting the primary regulator of the heat shock response—heat shock transcription factor 1 (HSF1) [14,85]. The anticancer molecule cantharidin significantly inhibited HSF1, its downstream HSP70 molecules, and its co-chaperone B-cell lymphoma 2 (Bcl-2)-associated anthanogene (BAG3). This inhibition was accompanied by increased cancer cell death [14]. Additionally, fisetin—a dietary flavonoid with anticancer potency—has been reported to promote the apoptosis of CRC cells by inhibiting HSF1 interaction with HSP70, thereby inhibiting the latter’s activity [85]. A novel HSP70 inhibitor (AP-4-139B) was recently identified that targets mitochondria of cancer cells but not of the normal cells, which proved to have inhibition potency and reduced toxicity [66]. Furthermore, there is increasing evidence of the upregulation of HSP70 expression and resistance to the HSP90 inhibitor, onalespib. Considering this, the efficacy of onalespib was tested in combination with the HSP70 inhibitor AT7519. AT7519 is a small-molecule inhibitor of CDK9—a catalytic subunit of the positive transcription elongation factor (P-TEFb)—that enhances the elongation of the HSP70 transcript. Using this combination resulted in promising preliminary clinical activity [86].

Furthermore, 2-Phenylethynesulfonamide (PES), also known as pifithrin-µ, is a well-known inhibitor of HSP70 that interacts explicitly with its substrate-binding domain (SBD) [87]. HSP70 binds to its client protein through a unique amino acid motif present on its SBD. PES competes with HSP70 client proteins and co-chaperones on SBD, thereby disrupting the folding of essential proteins required by cancer cells. PES combined with oxaliplatin chemotherapy showed potent anticancer effects on CRC cells [87]. Indeed, this combination was more effective with minimal side effects than when the elements were introduced separately [2]. Apoptozole is another recently identified small molecule that targets HSP70, specifically in CRC cells [14]. Apoptozole binds to the ATP binding pocket of HSP70 to inhibit its ATPase activity. Apoptozole has a potential anticancer effect on CRC cells both in vitro and in vivo. Lastly, MKT-077a cationic rhodacyanine derivative—has been reported to target the ATPase activity of HSP70 in CRC cells and was the first drug to reach the clinical trial stage [2]. However, the trial was terminated due to high drug toxicity. Another recently discovered antitumor alkaloid drug derived from the Liliaceae plant has been shown to inhibit mortalin-2 (mot-2) indirectly through the upregulation of UBX Domain Protein 2A (UBXN2A), which, in turns, inhibits mortalin [88].

Aptamers are the second group of HSP70 inhibitors that bind to different domains of HSP70 [2]. So far, few studies have considered HSP70 inhibitor aptamers that target CRC. Of the different aptamers tested, the A8 aptamer could bind to the extracellular domain of HSP70 and inhibit its oncogenic activity in a CT26 mouse colon cancer model [89].

The third group consists of monoclonal antibody-based inhibitors, including cmHsp70, which can bind to HSP70 on the membrane of CT26 cells by recognizing the specific amino acid sequence TKDNNLLGRFELSG (TDK). This binding induced antibody-dependent cellular cytotoxicity (ADCC) on bound cancer cells, reduced the tumor mass and increased OS in the CT26 model [90]. Unlike other commercial antibodies, cmHsp70.1 can bind and detect the expression of HSP70 in living tumor cells. However, A8 is favored over cmHSP70.1 because of its greater stability, solubility, and manufacturing simplicity [89]. 

Other HSP70 inhibitors have recently been identified that are not a member of any of the previous groups. One example is Pluronic^®^ which is a non-toxic sensitizing agent for the hyperthermia treatment of cancer [91]. Pluronic^®^ is a mixed copolymer of hydrophobic and hydrophilic polymers that have been shown to inhibit HSP70 expression in CRC cells under hyperthermia; thus, it seems that Pluronic^®^ enhances the toxicity of thermal treatment for CRC cells due to the inhibition of HSP70 activity [91]. Additionally, hexachlorophene—an antimicrobial compound and disinfectant—has been reported to target a specific member of HSP70 (GRP78) by binding to its SBD and inhibiting substrate binding [92,93]. Hexachlorophene treatment of CRC cells has been found to induce autophagy and increase apoptosis due to GRP78 inhibition.

Furthermore, n-3 PUFA docosahexaenoic acid (DHA)—a potent anticancer therapy—explicitly targetsGRP78. Previously, it was reported that DHA treatment induced apoptosis and reduced tumor growth in CRC [94,95]; however, few studies have investigated the DHA anticancer mechanism. It was recently reported that DHA reduced MEK/ERK pathway activation by inhibiting ERK phosphorylation. This inhibition caused the downregulation of GRP78 expression and altered its original location in the endoplasmic reticulum (ER) [94]. Together, these inhibitors and their role in CRC treatment show the need to understand the HSP70 oncogenic mechanism in CRC progression.

### 3.3. sHSP Inhibitors

sHSPs are ubiquitously expressed in different organisms, playing a significant role in cellular proliferation, differentiation, and degradation. sHSPs are not only involved in protein refolding but also play a role in attenuating the aggregation of proteins under stressful conditions [96]; therefore, they have been proposed for different types of cancer progression, including CRC, and different inhibitors are being tested for their potent anticancer effects [61]. Unlike other HSPs, sHSPs do not have ATP binding pockets to perform their function in an ATP-independent manner. Otherwise, they have three main domains; a structured α-crystalline domain (ACD), an amino-terminal region (NTR), and a carboxy-terminal region (CTR) [96]. In recent years, numerous anticancer drugs have been designed to specifically target different domains of sHSPs. These sHSP inhibitors are categorized according to four major groups: small-molecule inhibitors, aptamers, monoclonal antibodies, and antisense oligonucleotides (ASO) [2]. 

Different small-molecule inhibitors are being tested against HSP27. Quercetin—a member of the flavonoid group of polyphenols—has exhibited potent anticancer activity against primary colon cancer cells by binding to HSP27 and inhibiting its activity [97]. Nevertheless, no current clinical trials are testing quercetin’s effectiveness on humans [2]. In addition, YangZheng XiaoJi—a Chinese anticancer compound—can also inhibit HSP27 phosphorylation in different cancers, including CRC [98], by inhibiting HSP27 localization with caspase_9; the HSP27 function is inhibited in cancer cells through the inhibition of phosphorylation or its colocalization with caspase-9 [98]. Additionally, ovatodiolide (Ova) is a small-molecule inhibitor isolated from Anisomeles indica that has a potent anticancer stem cell (anti-CSC) effect on different cancers, including breast cancer and CRC [99,100]. In breast cancer cells, Ova reduced HSP27 expression to suppress tumor growth [100]. Although Ova has an anti-CSC effect on CRC cells, this effect was identified with a different mechanism in breast cancer [99]. Further studies are recommended to study its effect on HSP27 in CRC cells. Another naturally occurring anticancer agent is curcumin, the effect of which on CRC cells was tested after silencing HSP27. Interestingly, CRC cells lacking HSP27 exhibited resistance to curcumin treatment and, thus, reduced apoptosis; therefore, this study suggested that HSP27 is a potential target for curcumin in CRC [101]. A quinone-based pentacyclic derivative (3*S*,3′*R*) spiro[(hexahydropyrrolo[1,2-*a*]pyrazine-1,4-dione)-6,3′-(2′,3′-dihydrothieno[2,3-*b*]naphtho-4′,9′-dione)] (DTNQ-Pro) is a novel synthetic anti-cancer agent with broad-spectrum activity on different types of cancers, including CRC. Unlike other therapies, DTNQ-Pro did not reduce HSP27 expression, but caused its redistribution inside cancer cells to the cytoplasm compared to the perinuclear HSP27 in control cells [102].

The second inhibition approach focuses on monoclonal antibodies (mainly cetuximab), which block epidermal growth factor receptor (EGFR) activity [103]. Cetuximab sensitizes CRC cells to CPT-11—a chemotherapy drug—by suppressing HSP27 activity by targeting the Janus kinase/signal transducer and activator of transcription (JAK/STAT) signaling pathway. Interestingly, cetuximab could suppress HSP27 even in RAS- or BRAF-mutated cells considered resistant to cetuximab therapy. These findings may offer novel strategies for overcoming resistance to cetuximab in RAS- and BRAF-mutated CRC cells.

The third approach utilizes aptamers that can bind to HSP27 and inhibit its dimerization. The most well-known aptamers are PA11 and PA50, which bind to HSP27 oligomers and inhibit their tumorigenic effect [96]. The effect of these aptamers has only been studied in cancers other than CRC, including prostate cancer, small cell lung cancer (SCLC), and head and neck squamous cell carcinoma [2,96]. The effect of aptamer on CRC requires further study.

The final approach that was recently identified involves the antisense oligonucleotide (ASO). The ASO OGX427 has been shown to inhibit the mRNA expression of HSP27 [96]. ASO approaches have been extensively studied in patients with prostate, bladder, ovarian, breast, and non-small cell lung cancers [96]. In CRC, OGX427 activity was tested on the SW480 cell line to study the inhibition of gap junction intercellular communication (GJIC) formation mediated by HSP27. Notably, inhibition of HSP27 by OGX427 abolished the formation of GJIC and therefore altered the interaction of the CRC cell line with endothelial cells [104].

### 3.4. HSP60 Inhibitors

Targeting various HSPs for cancer chemotherapy has recently gained attention due to their significant role in CRC progression; however, few studies have targeted HSP60 activity and its co-chaperone HSP10 for CRC treatment due to their controversial role in different cancers. Nevertheless, there are two resources for HSP60 inhibitors—natural and synthetic—and, mechanistically, these inhibitors interact with ATP binding pockets or a specific cysteine residue in Hsp60 [105]. Various natural inhibitor compounds were tested against HSP60, including mizoribine, epolactaene, and myrtucommulone A (MC). Mizoribine was reported to inhibit the ATPase activity of HSP60, while epolactaene bound to the Cys442 residue of HSP60 to inhibit its activity [105]. Unfortunately, few synthetic compounds were able to target HSP60 activity, one of which was o-carboranylphenoxyacetanilide, but none of the above-mentioned inhibitors were tested on CRC cell lines. Overall, these studies have provided a better understanding of these inhibitors’ bioactivity and have therefore paved the way for testing on CRC.

Recently, another 24 different inhibitors were tested against HSP60 activity in CRC and showed significant inhibition of CRC cells compared to normal cells. Interestingly, the effect on CRC cell viability of those inhibitors was associated with the inhibition of expression of HSP60, thus, indicating that HSP60 might be a target for these inhibitors [106].

### 3.5. HSP110 Inhibitors

Only limited studies have focused on the role of HSP110 in CRC, and little research has targeted its activity. Gozzi and his colleagues identified two abiotic foldamers, 33 and 52, using chemical library screening to inhibit HSP110 functioning by targeting its NBD. These inhibitors were able to reduce CRC cell growth, as confirmed by an in vivo model. This study will open opportunities for discovering more molecules to target HSP110 for CRC treatment [107].

## 4. HSPs Promoting Therapy Resistance in CRC

One of the major concerns in CRC treatment is that cancer cells develop resistance to different therapies, causing reduced survival rates in CRC patients [108]. Nowadays, chemotherapy and radiotherapy are the commonly used treatments but increasing evidence has proved the role of HSPs in influencing CRC cells’ responses to these chemotherapies. The most important HSP in the development of resistance to chemotherapy is HSP90. HSP90 stabilizes several client proteins that play a role in cell proliferation [11]; thus, it allows cancer cells to develop resistance to different genotoxic treatments, such as 5-Fluorouracil (5-FU). In CRC, the inhibition of HSP90 following ganetispib treatment sensitized cancer cells to 5-FU, and HSP27 was observed to regulate the response of CRC cells to 5-FU [10].

Interestingly, resistance to 5-FU significantly correlated with the mRNA and protein levels of HSP27 in CRC cells. Furthermore, inhibition of HSP27 phosphorylation restored the sensitivity of CRC cells to chemotherapy [109]. Additionally, HSP110 overexpression is associated with increased resistance to both 5-FU and oxaliplatin [110]. Following the DNA damage induced by these chemotherapies, HSP110 translocate to the nucleus. Later, it stabilizes elements of the non-homologous end-joining (NHEJ) pathway, reducing DNA damage and killing CRC cells caused by 5-FU treatment [110]. Furthermore, a mutant form of HSP110 known as HSP110DE9 is specific to CRC patients with microsatellite instability (MSI). This mutant form is caused by skipping exon 9 and thus abrogating the form of HSP110. Patients with HSP110DE9 were reported to have a better response to 5-FU treatment [111].

Another example is HSP47, a collagen-specific chaperone [12], which was found to be overexpressed in CRC cell lines with established resistance to 5-FU. HSP47 increases the stability of AKT through the inhibition of PH-domain leucine-rich-repeat-containing protein phosphatase (PHLPP1)—a phosphatase that dephosphorylates AKT. The persistent activation of AKT, through HSP47 allows CRC cells to tolerate the environmental stress caused by 5-FU treatment [12]. These studies have proved the significant role of HSP inhibitors in combination with chemotherapy as a future approach for treating CRC patients resistant to these chemotherapies.

## 5. Conclusions

In conclusion, since the vast majority of HSPs have an oncogenic effect on CRC, their regulatory roles in activating various signaling cascades to promote CRC tumorigenesis, development, and metastasis have been extensively studied. Additionally, they have received attention for use as diagnostic, prognostic, and predictive biomarkers for CRC progression and metastasis and as targets for cancer therapy. This review sheds light on the functional mechanisms and the diagnostic and prognostic potentials of HSP90, HSP27, and HSP70 for CRC. Additionally, it has summarized current knowledge about the emerging therapies that target HSPs. Most anticancer therapies targeting HSPs in CRC are small-molecule inhibitors, but other approaches, such as aptamers and ASO, warrant further clarification. Sensitivity to different chemotherapies could be improved by combining them with various HSP inhibitors, proving the potential role of these inhibitors in the drug resistance caused by various CRC therapies. We recommend that researchers direct more attention towards combination therapy, including a targeted inhibitor of HSP, when designing a new anticancer therapeutic regimen.

## Figures and Tables

**Table 1:** HSPs localization, function, and their role prognostic markers in CRC. ER: Endoplasmic reticulum, ECM: Extracelluler matrix, F-FDG:Fluorodeoxyglucose (18F), EMT: Epithelial mesenchymal transition, OS: Overall survival, ROS: Reactive oxygen species, pT: post-therapy, RTK: Receptor tyrosine kinase, UPR: Unfolded protein response, RFS: relapse-free survival, NF-κB: Nuclear factor kappa B, IGFBP7: Insulin-like growth factor binding protein 7, AMPK: AMP-activated protein kinase, STAT3: Signal transducers and activation of transcription 3.

	HSPs	Cellular Location	Function	As Prognostic Markers
HSP90 family	HSP90	Cytoplasm, Cell Membrane, ECM	Stabilizes MutS p53 [19], promotes F-FDG accumulation [20], inhibits E-cadherin [21], mediates EMT [21]	Poor prognosis, an independent risk factor for OS [22]
	GRP94	ER	Activates immune cells [23]	Increased tumor size and pT stage [23]
	TRAP1	Mitochondrial Matrix	Protects the mitochondria from ROS accumulation [24]	Invasion and reduced OS [24]
HSP70 family	HSP70	Cytosol	Activates RTK [25], stabilizes β-catenin [26]	Prognostic marker in primary CRC [27]
	GRP75	Mitochondria	Promotes P53 retention [28] and Wnt/β-catenin pathway, EMT [26]	Poor OS and poor prognosis [26]
	GRP78	ER	Activates UPR [29]	
sHSP family	HSP27	Cytosol	EMT [30], dowregulates cell cycle-associated molecules [31], regulates Ca^2+^ influx [31], promotes the “*Warburg effect*” [32]	Primary tumor depth of CRC, and reduced recurrence-free survival [33]
	HSPB3	Cytosol		Poor RFS and OS [34]
	HSPB5	Cytosol	Inhibits NF-κB [35]	Tumor grade, potential prognostic maker [36]
HSP60 family	HSP60	Mitochondria	Enhances IGFBP7 activity [37], promotes adenine accumulation, activates AMPK [38]	Prognostic marker for the late stage of CRC and liver metastasis, early diagnostic marker [39]
HSP110		ER, cytosol, ECM	Activates STAT3 pathway [17] Favors anti-inflammatory macrophages [40]	Bad prognosis, poor OS, metastasis [9]

**Table 2:** Chemotherapies targeting HSPs inhibitors in CRC.

HSPs	HSP Inhibitors	Source	Mechanism of Action
HSP90	17-AAG	Geldanamycin derivative	Target the ATP binding pocket
	DDO-5936	Small-molecule inhibitor	Inhibits PPI with CdC37
	12c	Chemotherapies with resorcinol	Inhibits target-independent activation
HSP70	EGCG	Small-molecule inhibitor	Promotes Grp78 dimerization
	Quercetin	Flavonoid group of polyphenols	Reduces HSP70 expression
	Kahweol	Small molecule inhibitor	Reduces HSP70 expression
	Cantharidin	Small-molecule inhibitor	Inhibits HSF1
	Fisetin	Dietary flavonoid	Inhibits HSF1
	AP-4-139B	Small-molecule inhibitor	Target mitochondrial cancer cell
	AT7519	Small-molecule inhibitor	Inhibits CDK9
	PES	Small-molecule inhibitor	Interaction with SBD of HSP70
	Apoptozole	Small-molecule inhibitor	Target the ATP binding pocket
	MKT-077	Cationic rhodacyanine	Target the ATP binding pocket
	A8 aptamer	Aptamers	Binds to the extracellular domain of HSP70
	cmHsp70	Monoclonal antibody-based	Induces ADCC
	Pluronic	Sensitizing agent	Reduces HSP70 expression
	Hexachlorophene	Antimicrobial compound and disinfectant	Interaction with SBD of GRP78
	DHA	Polyunsaturated fatty acids	MEK/ERK pathway activation
sHSP	Quercetin	Flavonoid group of polyphenols	Binds to HSP27 and inhibits its activity
	YangZheng XiaoJi	Chinese anticancer compound	Inhibits HSP27 localization with caspases 9
	Ova curcumin	small-molecule inhibitor	anti-CSC effect on CRC
	DTNQ-Pro	Naturally according quinone-based pentacyclic derivative	HSP70 redistribution
	Cetuximab	monoclonal antibody-based	Inhibits JAK/STAT signaling pathway
	OGX427	ASO	Abolished the formation of GJIC
